# Incidence and risk factor profile for subcutaneous fat necrosis among newborns undergoing therapeutic hypothermia: a multi-center regional cohort

**DOI:** 10.1038/s41372-026-02772-0

**Published:** 2026-07-06

**Authors:** Brian H. Walsh, Hoda El-Shibiny, Sara Cherkerzian, Sara V. Bates, Munish Gupta, Anne Hansen, Emily Herzberg, Janet S. Soul, Terrie Inder, Mohamed El-Dib

**Affiliations:** 1 Department of Neonatology, Cork University Maternity Hospital, Cork, Ireland; 2 Division of Newborn Medicine, Department of Pediatrics, Brigham and Women’s Hospital, Boston, MA USA; 3 Harvard Medical School, Boston, MA USA; 4 Division of Newborn Medicine, Mass General Hospital for Children, Boston, MA USA; 5 Department of Neonatology, Beth Israel Deaconess Hospital, Boston, MA USA; 6 Division of Newborn Medicine, Boston Children’s Hospital, Boston, MA USA; 7 Department of Neurology, Boston Children’s Hospital, Boston, MA USA; 8 Center for Neonatal Research, Children’s Hospital of Orange County, Orange County, CA USA; 9 Irvine—College of Medicine, University of California, Irvine, CA USA

**Keywords:** Paediatrics, Paediatric neurological disorders, Outcomes research

## Abstract

**Objective:**

To determine the incidence, risk factors, and outcomes associated with subcutaneous fat necrosis (SCFN) in a regional cohort of newborns treated with therapeutic hypothermia (TH) for neonatal encephalopathy (NE).

**Study design:**

Data from a regional Neonatal Encephalopathy Registry was retrospectively analysed. All newborns treated with TH between 2018 and 2022 were included. The primary outcome was the incidence of SCFN. Secondary outcomes included associated risk factors and complications.

**Results:**

SCFN occurred in 4% of newborns (20/499) undergoing TH. Factors associated with SCFN included elevated maternal BMI, maternal preeclampsia/hypertension, pyrexia during labor, earlier initiation of active cooling, thrombocytopenia, and blood product administration. Half of the SCFN cases developed hypercalcemia. SCFN was associated with longer hospital stay (median 11.5 vs. 8 days, *p* = 0.007).

**Conclusion:**

In this large, contemporary cohort, SCFN occurred in 4% of newborns with NE treated with TH. Enhanced monitoring and risk stratification may facilitate early diagnosis and improve management outcomes.

## Introduction

Subcutaneous fat necrosis (SCFN) is known to affect newborns who received therapeutic hypothermia (TH) for neonatal encephalopathy (NE). SCFN is typically a self-limited panniculitis, but it can result in discomfort, scarring, and hypercalcemia necessitating treatment. Histologically, it is characterized by necrotizing granulomas within adipose cells [1]. The exact pathophysiological mechanism is not fully understood, but it is thought to have a multifactorial etiology with a reduction in perfusion, leading to hypoxic cellular damage of the subcutaneous adipose tissue [2, 3]. Newborns are at a higher risk of SCFN than adults. This may be due to neonatal adipocytes being more susceptible to cold stress because of a higher proportion of saturated fatty acids than adult adipocytes [4, 5].

SCFN was rarely reported in the neonatal literature prior to the introduction of TH, and was limited to sporadic case reports [6]. Even during the original randomized controlled trials of TH, only a single case of SCFN was documented in the NICHD trial, and none of the other original trials reported SCFN [7]. However, as the use of TH has increased, data have emerged on the increased identification of SCFN in newborns treated with TH [8–12]. These data are mostly case series and retrospective small cohorts, limiting the ability to fully describe SCFN in this patient population, including both the incidence and risk factors for its development.

The aim of this study was to investigate the incidence, risk factors, and early natural history of SCFN among a large regional cohort of newborns with NE treated with TH.

## Methods

This is a retrospective analysis of data from a Regional Neonatal Encephalopathy Registry. In 2016, the Academic Medical Center Patient Safety Organization (AMC PSO), a component entity of the Risk Management Foundation of the Harvard Medical Institutions Incorporated (CRICO), convened a Task Force to develop and publish regional TH guidelines (CRICO TH Guidelines available at: https://www.rmf.harvard.edu/-/media/Files/_Global/KC/PDFs/Guidelines/crico_neonates.pdf). These guidelines include criteria for when TH is indicated, and criteria for when TH may be considered, which includes borderline eligibility criteria. The application of TH for borderline eligibility or milder encephalopathy is not standard of care, however it acknowledges the well documented therapeutic drift that is occurring internationally [13–15]. Following publication of the guidelines, the Task Force established an IRB-approved prospective data repository to monitor screening for neonatal encephalopathy and the application of TH for at-risk newborns in the CRICO network. Fourteen centers in Massachusetts, either affiliated with Harvard Medical School and/or receiving medical malpractice via the CRICO insurance program, agreed to contribute data to the registry. The 14 centers include; 4 cooling sites (1 specialty children’s hospital and 3 delivery hospitals providing TH), 9 referring delivery hospitals, and 1 transport service. The registry was designed to assess the impact of the established guidelines, provide information about the current epidemiology of NE, and allow for the identification of knowledge gaps and areas that would benefit from quality improvement efforts. The registry was launched in 2018, and we have previously published details about the registry [16], including the institutions involved, and the REDCap data instruments developed and used to generate the registry database [17, 18]. This study followed the STROBE reporting guideline for cohort studies [19].

### Data collection

The NE Registry Database is composed of the following data domains: Demographics, Maternal History, Delivery History, Newborn Delivery, Neurology, Therapeutic Hypothermia, Neuromonitoring, Short Term Outcome, and Side-Effects of TH. Over 180 data points are collected for each newborn evaluated for TH. Full details of the case report forms are available in our previous publication detailing the establishment of the registry [16].

For the current study, eligibility was limited to those newborns in the registry treated with TH between 2018 and 2022 at one of the 4 cooling centers. TH eligibility criteria across these sites are based on that published in the regional TH Guidelines mentioned above [16]. Across this collaborative network, the severity of encephalopathy was determined using one of three standardized neurological exams; the modified Sarnat exam, and two adapted exams, which are local modifications that provide a numerical score rather than a classification of mild, moderate, or severe. These numerical scores are termed the Neonatal Encephalopathy Score (NES), which is used in one site, and the Brief Neonatal Encephalopathy Score (BNES), used in two sites (Both detailed in Supplementary file). We have previously published on both of these scores, and have demonstrated their association with grade of encephalopathy [20, 21]. While similar, these numerical scores are not identical, preventing the direct comparison of NE severity between sites. Therefore, the documentation of all neurological exams assigning a numerical score was reviewed, and where possible a clinical grade (mild, moderate or severe) based on the modified Sarnat was assigned, to allow comparison of NE severity between sites.

All centers provide whole-body TH, using one of three commercially available cooling devices (Blanketrol III is used by two sites, Criticool used by one site, and Arctic Sun used by one site). TH was ideally initiated within 6 h of birth, continued for 72 h, and followed by slow re-warming, per each institution’s guideline for TH. Multichannel EEG (cEEG) is routinely applied for those undergoing TH across the participating centers, and MRI is performed following rewarming. cEEG severity was taken from the clinical report of the first 24 h of recording. The presence of hypoxic ischemic MRI abnormality was taken from the neuroradiologists’ clinical report.

The regional TH guidelines specifically identify the need to monitor for SCFN and associated hypercalcemia. Following their launch, CME-accredited educational sessions were offered to participating centers to familiarize clinicians with the guidelines, including the need to monitor for SCFN. For the registry, diagnosis of SCFN was made by the clinical team and entered as a pre-specified data point. Hypercalcemia was defined as a lab calcium of >10.5 mg/dL or an ionized calcium >1.38 mmol/L [22, 23].

### Data extraction

Newborns treated with TH with and without SCFN were identified from the data registry. Data extracted for analysis of potential risk factors and associations included details of delivery and cooling site, newborn demographics (gestational age, sex, birth weight and centile), ante-natal history (maternal medications, chronic illness, thyroid disease, gestational diabetes, hypertension and pre-eclampsia), peri-partum course (septic risk factors, chorioamnionitis, meconium stained amniotic fluid, abnormal fetal heart traces and delivery complications), method of delivery and resuscitation details, and relevant post-natal data on severity of encephalopathy, TH, medications, hematological studies and transfusions, and outcomes. These data points were chosen for analyses based upon previously published literature [1, 6, 24, 25]. Among those who developed SCFN, additional data were extracted from the electronic health records (EHR), including the day of life when the SCFN was diagnosed, details of investigations performed (e.g., biopsies, lab results), whether hypercalcemia developed [22], and if so, details on any treatment (e.g., diuretic use, low calcium formula, or steroids) provided.

### Statistical methods

For all infants treated with TH in the registry, we compared those with and without a diagnosis of SCFN (SCFN present vs. absent). Descriptive statistics for identified variables were reported by SCFN status. The mean and standard deviation were reported as the central tendency of the distribution unless non-normally distributed, for which the central tendency was reported as the median (IQR). Differences in variables by group status were tested using chi-square test (or Fisher’s exact test where cell size <5) and Wilcoxon rank-sum tests, for categorical and continuous variables, as appropriate. Comparison of the clinical stage of NE between those with SCFN present vs. absent was limited to data from 2020 to 2022 inclusive, following the modification to the data registry (detailed in Data Collection section above). Statistical significance was determined using *p*  <  0.05. Analyses were exploratory and were not adjusted for multiple comparisons [26]. All analyses were performed using SAS v9.4 software (SAS Institute, Cary, NC).

## Results

Four hundred and ninety-nine newborns receiving TH were entered into the registry between 2018 and 2022. Twenty newborns were reported to have SCFN, resulting in an incidence of 4%. The median age of identifying SCFN was day of life 4 (IQR, day 2–5), and the most common location was over the back (60%). There was no difference in the incidence of SCFN between cooling centers (Center 1, 5.3% [6/114]; Center 2, 2.6% [2/76]; Center 3, 3.6% [7/193]; and Center 4, 4.3% [5/116]; *p* = 0.82), nor with being out-born (35% of SCFN present vs. 42.4% of SCFN absent, *p* = 0.51).

Table 1 shows demographic and perinatal variables among the two groups. Sex, birth weight, and gestational age were not associated with the development of SCFN. Ante-natal risk factors associated with SCFN development included maternal pre-pregnancy body mass index (BMI) ≥ 30 (SCFN present vs. absent; 25% vs. 10%, *p* = 0.03), and pre-eclampsia (SCFN present vs. absent; 25% vs. 10%, *p* = 0.03). Additionally, SCFN was associated with maternal hypertension during pregnancy (SCFN present vs. absent; 35% vs 15.7%, *p* = 0.02). This association was not specific to chronic or gestational hypertension. The only perinatal factor associated with SCFN was maternal pyrexia >101 °F (SCFN present vs. absent; 25% vs 9.6%, *p* = 0.03) and chorioamnionitis (SCFN present vs. absent; 35% vs 17.7%, *p* = 0.05). There was no association between SCFN and the method of delivery, presence of meconium, or degree of resuscitation (Table 1).

**Table 1:** Neonatal demographic details, and perinatal risk factors for SCFN.

	Subcutaneous fat necrosis present (*n* = 20)	Subcutaneous Fat Necrosis Absent (*n* = 479)	*P* value
**Demographics**			
Female Sex, n (%)	12 [60]	194 (40.5)	0.08
Singleton, n (%)	20 (100)	460 [96]	0.38
Gestation (median, IQR)	39.1 (37.0–40.4)	39.3 (37.5–40.1)	0.87
Birth weight, grams (median, IQR)	3265 (2920–3732)	3200 (2830–3580)	0.49
Birth weight z-score, (mean ± SD)	0.3 ± 1.1	–0.1 ± 1.2	0.28
Birth weight category, n (%)			0.25
SGA	1 (5)	58 (12.1)	
AGA	15 [75]	373 (77.9)	
LGA	4 (20)	9.8 [47]	
Outborn, n (%)	7 (35)	203 (42.4)	0.51
**Maternal risk factors**			
Pre-pregnancy BMI > 30, n (%)	5 (25)	48 (10)	0.03
Diabetes, n (%)	4 (20)	95 (19.8)	0.98
Pre-gestational DM Gestational DM	0 (0) 4 (20)	13 (2.7) 82 (17.1)	
HTN, n (%)	7 (35)	75 (15.7)	0.02
Pre-gestational HTN	3 (15)	25 (5.2)	
Gestational HTN	4 (20)	49 (10.2)	
Preeclampsia, n (%)	5 (25)	48 (10)	0.03
Thyroid disease, n (%)	1 (5)	46 (9.6)	0.49
**Maternal medications**			
Antidepressants, n (%)	2 (10)	69 (14.4)	0.58
Anxiolytics, n (%)	0 (0)	9 (1.9)	0.53
Anti-hypertensives, n (%)	2 (10)	22 (4.6)	0.26
Insulin, n (%)	2 (10)	26 (5.4)	0.38
No medications, n (%)	14 (70)	241 (50.3)	0.08
Recreational drugs, n (%)	0 (0)	13 (2.7)	0.45
**Delivery and resuscitation**			
Clinical chorioamnionitis, n (%)	7 (35)	85/477 (17.7)	0.05
Maternal temp >101 ﹾF, n (%)	5 (25)	46 (9.6)	0.03
Meconium stained fluid, n (%)	9 (45)	166/477 (34.7)	0.35
Method of Delivery, n (%)			
Vaginal	9(45)	215 (44.9)	0.99
Forceps	1 (5)	28 (5.8)	0.87
Vacuum	3 (15)	64 (13.4)	0.83
Elective C-Section	0 (0)	11 (2.3)	0.49
Emergency C-section	11 (55)	243 (50.7)	0.70
Resuscitation required, *n* (%)	17 (85)	424 (88.5)	0.63
Duration of PPV, min (mean ± SD)	4.5 ± 2.6	4.0 ± 3.9	0.25
Intubated at delivery, n (%)	8 (40)	173 (36.1)	0.49
Chest compressions, n (%)	5 (25)	66 (13.8)	0.29
Epinephrine, n (%)	3 (15)	38/478 (7.9)	0.44
1 min Apgar, (median, IQR)	2 (1–2)	2 (1–3)	0.10
5 min Apgar, (median, IQR)	5 (3–7)	6 (4–7)	0.24
10 min Apgar, (median, IQR)	7 (5–7)	7 (5–8)	0.57
UA pH, (mean ± SD)	7.0 ± 0.1	7.0 ± 0.2	0.63
UA BD, (mean ± SD)	14.1 ± 5.0	12.3 ± 5.7	0.14
1st post-natal pH, (mean ± SD)	7.2 ± 0.1	7.2 ± 0.1	0.49
1st post-natal BD, (mean ± SD)	14.1 ± 5.3	12.4 ± 5.6	0.22

Missing data: Denominator detailed if missing data for specific variable reported as frequency or proportion.

Variables reported as mean or median with missing data, and number of data points missing- 10 min Apgar, SCFN absent *n* = 134, SCFN present *n* = 3; UA pH, SCFN absent *n* = 106, SCFN present *n* = 2; UA BD, SCFN absent *n* = 148, SCFN present *n* = 5; 1st post-natal pH, SCFN absent *n* = 69, SCFN present = 3; 1st post-natal BD, SCFN absent *n* = 98, SCFN present *n* = 6.

*SGA*small for gestational age ( < 10%ile), *AGA* appropriate for gestational age, *LGA* large for gestational age ( > 90%ile), *HTN*hypertension, *PPV* positive pressure ventilation, *UA* umbilical artery, *BD* base Deficit.

Table 2 provides details on the severity of NE, and the treatment provided. Ninety-one percent (458/499) of newborns had a standardized neonatal encephalopathy exam documented, with 197 having the NES, 153 the BNES, and 107 having a modified Sarnat as the primary exam performed. A clinical grade of NE (mild, moderate or severe) was able to be assigned in 76% (381/499). There was no difference in the clinical stage of NE between those who did and did not develop SCFN (*p* = 0.98). Ninety-eight percent (*n* = 491) of newborns had cEEG performed. There was no difference in cEEG background pattern (*p* = 0.91), the presence of seizures (*p* = 0.41), or the administration of anti-seizure medications between those who did and did not develop SCFN (*p* = 0.74).

**Table 2:** Details on therapeutic hypothermia, grade of encephalopathy, and short-term outcome among those that did and did not develop SCFN.

	Subcutaneous fat necrosis present (*n* = 20)	Subcutaneous fat necrosis absent (*n* = 479)	
**Degree of encephalopathy**			
Standardized NE exam documented, n (%)	20 (100)	438 (91.4)	0.17
*Clinical Stage of NE, n (%)			0.98
Mild	7 (35)	131/361 (36.2)	
Moderate	10 (50)	180/361 (49.8)	
Severe	3 (15)	50/361 (13.8)	
cEEG background grade, n (%)			0.91
Not classified	4 (20)	94/468 (19.6)	
Normal	4 (20)	90/468 (18.8)	
Mild	7 (35)	183/468 (38.2)	
Moderate	3 (15)	77/468 (16.1)	
Severe	2 (10)	24/468 (5)	
Seizures, n (%)			0.41
Electrical Only	1 (5)	34 (7.1)	
Electroclinical	0 (0)	35 (7.3)	
Abnormal MRI, n (%)	14 (70)	246/464 (51.4)	0.14
Abnormal neurological exam at discharge, n (%)	3 (15)	50/476 (10.4)	0.52
**Therapeutic Hypothermia**			
Passive Cooling, % (n)	95 (19)	88.1 (422/477)	0.65
Age passive TH started, (median, IQR)	0.5 (0.3–1.3)	0.8 (0.4–1.8)	0.07
Age active TH started, (median, IQR)	2.5 (1.4-3.7)	3.9 (2.6–5.3)	**0.002**
Age reached 33–34 C, (median, IQR)	3.3 (2.4–4.2)	4.4 (3.3–5.8)	**0.002**
Age Rewarming commenced, (median, IQR)	75.0 (73.4–76.3)	76.2 (74.7–77.8)	**0.03**
Age Rewarming Completed, (median, IQR)	84.7 (82.6–89.2)	86.6 (83.8–89.5)	0.23
Total duration Cooled, hrs (median, IQR)			
Time passive TH started to Rewarming completed	84.4 (81.3–88.6)	85.5 (82.3–87.9)	0.78
Time active TH started to Rewarming completed	82.6 (79.8–85.2)	83.0 (79.3–85.0)	0.9
Early Exit, *n* (%)	1 (5)	25 (5.2)	0.96
**TH details among Outborn newborns (n** = **210)**			
Passive Cooled in transport, *n* (%)	2 (28.6)	98 (48.3)	0.31
Active Cooled in transport, *n* (%)	5 (71.4)	100 (49.3)	0.25
Temperature (°C) on leaving referral center (median, IQR)	34.1 (33.4–34.7)	34.0 (33.5–35.6)	0.84
Temperature (°C)on arriving TH center (median, IQR)	33.5 (33.4–33.5)	33.6 (33.5–34.5)	0.10
**Medications**			
Received ASMs, n (%)	1 (5)	79 (16.5)	0.17
Received sedation, n (%)	20 (100)	469 (97.9)	0.83
Morphine infusion	19 (95)	442 (92.3)	0.65
Fentanyl infusion	2 (10)	33 (6.9)	0.59
Midazolam infusion	3 (15)	26 (5.4)	0.07
Inotropes, n (%)	6 (30)	83 (17.3)	0.15
**Clinical Course**			
Required intubation any time, n (%)	9 (45)	219/478 (45.7)	0.94
DOL first enteral feed, (median, IQR)	6 (5.0–9.5)	6.0 (5.0–7.0)	0.16
Length of stay, (median, IQR)	11.5 (7.5–27.5)	8 (6-13)	**0.007**
Death, n (%)	0 (0)	18/478 (3.8)	0.36

Missing data: *Data on infants from 2020 to 2022 inclusive due to change in register for this variable (*n* = 294 total)[detailed in methods].

Denominator detailed if missing data for specific variable reported as frequency or proportion.

Variables reported as mean or median with missing data, and number of data points missing- Age Passive TH started, SCFN absent *n* = 77, SCFN present *n* = 1.

*NE* neonatal encephalopathy, *cEEG* continuous multichannel electroencephalography, *MRI* magnetic resonance imaging, *TH* therapeutic hypothermia, *ASM* anti-seizure medication, *DOL* day of life, *NS* non significant.

Values in bold are considered signifi cant results.

Those who developed SCFN had active TH initiated earlier, achieved target temperature earlier, and had rewarming initiated earlier compared to those without SCFN. There was no difference in exposure to passive cooling (*p* = 0.65) or total duration of cooling between those who did versus did not develop SCFN (Table 2).

There was increased thrombocytopenia during TH (SCFN present vs. absent; 65% vs. 28% *p* < 0.001), and a higher frequency of blood product use (SCFN present vs. absent; 50% vs 26.7%, *p* = 0.02) in those with versus without SCFN. Specifically, the frequency of platelet transfusion (*p* = 0.01), fresh frozen plasma (*p* = 0.01), and cryoprecipitate (*p* = 0.01) were all higher in those with SCFN. There was no difference in the frequency of major hemorrhages between the groups (Table 3).

**Table 3:** Hematological parameters in those who did and did not develop SCFN.

	Subcutaneous fat necrosis present (*n* = 20)	No subcutaneous fat necrosis absent (*n* = 479)	
Hemorrhage requiring immediate transfusion, n (%)	1(5)	4/477 (0.8)	0.07
**Thrombocytopenia during TH** (platelet count <150,000), n (%)	**13 (65)**	**136/473 (28.4)**	**0.0002**
Among those with thrombocytopenia Lowest Platelet, (median, IQR)	81,000 (41,500-120,500)	83,000 (54,000–120,500)	0.90
Highest PT, (median, IQR)	19.1 (16.3–25.4)	18.0 (16.3–21.0)	0.42
Highest PTT, (median, IQR)	51.4 (37.6–96.5)	49.1 (41.0–65.5)	0.72
Lowest Fibrinogen, (median, IQR)	201.5 (115–276)	172.0 (141–209)	0.90
Lowest Hct (median, IQR)	37.9 (35.1–42.2)	40.5 (34.6–46.6)	0.42
**Received any blood product**, n (%)	**10 [50]**	**128 (26.7)**	**0.02**
Received PRBC	3 (15)	61 (12.7)	0.77
**Received platelets**	**5 (25)**	**40 (8.4)**	**0.01**
**Received FFP**	**8[40]**	**85 (17.7)**	**0.01**
**Received cryoprecipitate**	**5 (25)**	**41 (8.6)**	**0.01**

*PT* prothrombin time, *PTT* partial thromboplastin time, *Hct* hematocrit.

Missing data: Denominator detailed if missing data for specific variable reported as frequency or proportion.

Variables reported as mean or median with missing data, and number of data points missing- Highest PT, SCFN absent *n* = 198, SCFN present *n* = 10; Highest PTT, SCFN absent *n* = 198, SCFN present *n* = 10; Lowest Fibrinogen, SCFN absent *n* = 201, SCFN present *n* = 10; Lowest Hct, SCFN absent *n* = 198, SCFN present *n* = 10.

Values in bold are considered signifi cant results.

Fifty percent of newborns with SCFN (10/20) had documented hypercalcemia (Table 4). The highest calcium was recorded on median day 8 of life (IQR 6.5–12.75). Two cases of hypercalcemia were treated, consisting of a low calcium formula and diuretics.

**Table 4:** Details of cases with SCFN.

Demographics Sex, GA, Bwt	Pregnancy complications	Blood Products received	Thrombocytopenia (If yes, lowest platelet)	DOL SCFN Noted	Location SCFN	Description	Hypercalcemia If yes; DOL highest Ca Highest Ca	Treatment of hypercalcemia
Male, 37.2 wk 3060 g Moderate NE	No	PRBC Cryoprecipitate	Yes, 90,000	4	**	Mild edema	No	N/A
Male. 37.0 wk 3610 g Severe NE	Gest-HTN. GDM	PRBC. Platelet. FFP	Yes, 72,000	5	Abdomen	Area of necrosis and erythema	No	N/A
Male. 40.3 wk . 4515 g. Moderate NE	No	FFP	Yes, 140,000	3	Back, buttocks, thighs, and unilateral arm	Indurated, tender, hard area	No	N/A
Female. 41.7 wk. 3930 g. Moderate NE	Chorioamnionitis	No	No	5	Unilateral leg	A small, oval abrasion	No	N/A
Female. 41.1 wk. 4359 g. Moderate NE	No	Platelet. FFP	Yes, 114,000	6	**	**	No	N/A
Female. 40.0 wk. 3140 g. Mild NE	Chorioamnionitis	No	Yes, 121,000	4	Back	reddened oval raised patch, palpable induration underneath	Yes. Day 7. 10.8 mg/dL	No
Male. 38.1 wk. 4775 g. Moderate NE	BMI > 30. Chorioamnionitis	FFP	Yes, 53,000	6	Back	dark reddened area. Tender	Yes. Day 4. 11.3 mg/dL	No
Female. 39.5 wk. 3570 g. Mild NE	No	No	Yes, 66,000	5	Back	Redness, swelling, firmness	Yes. Day 8. 11.4 mg/dL	No
Female. 37.0 wk. 3100 g. Mild NE	Gest-HTN. PET	No	No	2	Lower back, sacral area	Erythematous, area	No	N/A
Male. 39.8 wk. 3540 g. Moderate NE	HTN. PET. BMI > 30. Chorioamnionitis	No	No	2	Back	Diffuse area of erythema, edema, firmness and nodularity	Yes. Day 20. 11 mg/dL	No
Female. 41.3 wk. 3850 g. Mild NE	HTN. Chorioamnionitis	Cryoprecipitate	Yes, 138,000	2	Unilateral leg	Firm, non-tender, mildly erythematous	No	N/A
Female. 38.4 wk. 2960 g. Mild NE	Chorioamnionitis	No	No	5	Back	Erythematous, mildly indurated and slightly elevated	No	N/A
Male. 35.4 wk. 2080 g. Severe NE	No	Platelet. FFP. Cryoprecipitate	Yes, 49,000	**	**	**	**	**
Female. 36.3. 2020 g. Moderate NE	Gest-HTN. GDM. PET	FFP. Cryoprecipitate	Yes, 12,800	2	Back	Nodules with erythema	Yes. Day 8. 11.4 mg/dL	No
Male. 35.5 wk. 2340 g. Moderate NE	PET. BMI > 30	Platelet. FFP	Yes, 30,000	5	Back, and upper thorax	Palpable nodules	Yes. Lab Ca not available. Highest ionized Ca 1.44	No
Male. 36.5 wk. 2630 g. Moderate NE	Gest-HTN. GDM. PET. BMI > 30	PRBC. Platelet. FFP Cryoprecipitate	Yes, 10,000	3	Back and coccyx	Hardened, reddened nodules	Yes. Day 9. Lab Ca not available. Highest ionized Ca 1.46	No
Female. 39.3 wk. 3615 g. Moderate NE	No	No	No	**	**	No skin description documented	No	N/A
Female. 38.8 wk. 2945 g. Severe NE	HTN. GDM. BMI > 30. Chorioamnionitis	No	No	34	Back	No further details documented	Yes. Day 29. 11.9 mg/dL	Low Ca Formula and diuretics
Female. 40.5 wk. 2895 g. Mild NE	No	No	No	3	Back and elbows bilateral	**	Yes. Day 6. 11.3 mg/dL	Low Ca Formula and diuretics
Female. 41.4 wk. 3390 g. Mild NE	No	No	Yes (**)	2	Back	Hardened area of erythema. Over time, the central area of necrosis was noted to become fluctuant and soft.	Yes. Day 12. 10.9 mg/dL	No

Missing data: **=data not documented in EHR.

*wk* week, *g* grams, *BMI* body mass index, *HTN* hypertension, *DM* diabetes mellitus, *PET*pre-eclampsia, *FFP* fresh frozen plasma, *PRBC* packed red blood cells, *N/A* not applicable.

## Discussion

In a large cohort of newborns treated with TH, the incidence of SCFN was 4%. The only maternal factors associated with the development of SCFN in this cohort were maternal hypertension/PET, BMI > 30, or pyrexia, and in newborns, earlier initiation of cooling, and receipt of blood products (specifically platelets, fresh frozen plasma, and cryoprecipitate). Sixty-five percent of newborns with SCFN had thrombocytopenia, and 50% developed hypercalcemia.

The incidence of SCFN in the general population of newborns is unknown, while the incidence among newborns with NE who undergo TH has been reported between 1 and 7% [9–11]. The TOBY registry was established following the TOBY trial, to assess the implementation of TH in the real-world setting [13, 27]. It is the largest dataset (*n* = 1239) to report an association between TH and SCFN, reporting an incidence of 1% [10]. It was also the first dataset to report this association. Therefore, it is likely that clinicians contributing data were less aware of the possibility of SCFN and may not have detected all cases. More recently, Courteau et al. reported a higher incidence of 7%, but with a smaller sample size and wider confidence interval [12]. The present cohort’s incidence of 4% lies midpoint between this previously reported range. The generalizability of this result is further supported by the narrow range of incidences reported across all participating cooling sites (2.5–4.7%). This is despite the four sites using three different cooling devices (Blanketrol III, Criticool, and Arctic Sun) and having some variations in local care practices.

Previous reports have identified several antenatal risk factors associated with SCFN, including gestational diabetes, maternal thyroid disease, and recreational drug use in pregnancy [1, 24, 28, 29]. However, none of these risk factors were found to be significant in the present study. The current cohort differed from these previous cohorts as it was limited to newborns treated with TH, as opposed to all newborns who developed SCFN. As such this was a high-risk population, with a higher incidence of pregnancy complications than the general population. For example, 20% of those with SCFN and 17.1% of those without SCFN had a pregnancy complicated by gestational diabetes within this cohort, compared to 6.9% of US and Canadian pregnancies in general [30]. Therefore, it is likely that the risk factor profile among this high-risk population is different to that of the general population. The antenatal and peripartum risk factors significantly associated with SCFN in the current study were obesity, pre-eclampsia, hypertension (gestational or pre-gestational), and maternal pyrexia. While these conditions may influence the vascular bed, adipocytes, and inflammatory pathways, the precise pathological mechanisms associated with these risk factors and the development of SCFN remain unclear.

Similarly, we report a few postnatal factors for the development of SCFN in our cohort. There was no association with sex, multiplicity, gestational age, severity of encephalopathy, seizures, or biochemical markers of asphyxia [11, 25, 28]. While we did not find an association between the development of SCFN and total duration of cooling, we did find an association between SCFN and the earlier initiation of active cooling. A potential explanation for this observed association is that tissue perfusion from the initial asphyxial insult may be more compromised at an earlier time point, and thereby exacerbate the impact of earlier cooling. Regardless, the benefits of the timely initiation of TH for those eligible to ensure optimum neuroprotection likely outweigh the risk of SCFN, and take precedence.

Blood products, specifically platelets, fresh frozen plasma, and cryoprecipitate, were more commonly administered to those with SCFN versus those without. These blood products were administered prior to the detection of SCFN. Blood products have not previously been identified as a risk factor for the development of SCFN. Such products contain numerous biologically active substances that have been associated with micro-clot development, alterations in vascular tone and growth, and immunomodulatory and inflammatory effects [31, 32]. It is therefore plausible that these effects may exacerbate reduced skin perfusion and the inflammatory response. However, this registry can only describe an association, and cannot determine if the products are themselves a risk factor or are a surrogate for an alternate risk factor.

Among those who developed SCFN, the median age of identification was day 4, with the back being the most common location. These findings are consistent with previous studies, which have reported that the majority of cases are identified in the first 7 days after birth [25, 28, 29], with a similar pattern of distribution [33]. SCFN was associated with the presence of thrombocytopenia and hypercalcemia. Both associations have been previously reported. Thrombocytopenia has been postulated to be secondary to the sequestration of platelets in the subcutaneous tissue [34], while hypercalcemia is thought to occur secondary to activated macrophages in fat granulomas releasing 1 alpha-hydroxylase which converts vitamin D to the active form [25, 29]. Of those who developed hypercalcemia, two required treatments, consisting of low calcium formula and diuretics. No newborn required additional management of hypercalcemia. Newborns who developed SCFN had a longer hospital stay than those who did not. This registry can only demonstrate associations and cannot determine causality, however given that markers of NE severity were comparable between groups, it is possible that the delayed discharge was secondary to the SCFN, its complications and management. This is relevant as it demonstrates the significant impact and cost associated with this condition.

There are several strengths of this study. First, this represents one of the largest cohorts of newborns with NE treated with TH with data collected regarding SCFN. It is a contemporary cohort recruited across multiple sites, and as such is highly generalizable and reflects current neonatal encephalopathy profiles and management strategies. Secondly, the importance of SCFN was identified at the inception of our database. Due to the infrequency of SCFN, only data from a large database can be used to determine the incidence and risk factor profile for SCFN among newborns undergoing TH. Data on SCFN was gathered prospectively, limiting the potential for under-diagnosis of SCFN within our cohort. This study also has limitations, primarily that we do not have data on the clinical course after hospital discharge. It is possible that an infant developed SCFN after discharge. However, this is likely a relatively rare event, given that the typical onset of SCFN is within the first week of life (consistent with our own results), during which these newborns typically remained inpatients. A second potential limitation is that one of the cases of SCFN had a gestation of 35.5 weeks. While we found no association between gestation and SCFN, given the small proportion of infants less than 37 weeks in our cohort, it raises the question whether late preterm infants have an increased risk of SCFN. Faix et al. recently published their RCT of TH in late preterm infants (33-35 + 6 wk), within which they reported on the occurrence of SCFN in their cohort [35]. They found no increased association among late preterm infants, with no cases of SCFN reported. While this publication has caused debate about how to manage these infants [36], from our perspective it reassuringly does not demonstrate any increased risk of SCFN among them. However determining how best to manage infants at this gestation is beyond the scope of this manuscript, and as the recent AAP TH guidelines recommend when considering TH at 35-35 + 6 weeks is best determined in discussion with the parents [37].

This work highlights that SFNC is a complication which all providers who care for infants undergoing TH will encounter and must be aware of. Its incidence was consistent across all participating sites in this cohort, and occurred independent of whole-body cooling device used or severity of NE. Practitioners should routinely monitor newborns undergoing TH for early dermatological signs of SCFN, especially on the back or pressure-bearing areas during the first days of life. Additionally, routine screening for associations of SCFN, such as hypercalcemia and thrombocytopenia, coupled with multidisciplinary care coordination, can facilitate timely diagnosis and management. While we recommend standardizing clinical guidelines for early detection and management of SCFN, more research is needed to explore any modifiable risk factors.

## Conclusion

In a large contemporary cohort of newborns with NE treated with TH we report a 4% incidence of SCFN. The antenatal and intrapartum factors that were associated with SCFN development were elevated maternal BMI, maternal pre-eclampsia/hypertension, and maternal pyrexia during labor. The postnatal factors associated were earlier initiation of active cooling and receipt of blood products. The median age of SCFN identification was 4 days. This is relevant as early identification is important to ensure appropriate screening for complications and arranging outpatient follow-up. Ongoing efforts to prevent this complication are worthy of further study.

## Supplementary information

Supplemental Material

## Data Availability

The datasets analysed during the current study are available from the corresponding author on reasonable request.
