# Supplementary material for: Incidence and risk factor profile for subcutaneous fat necrosis among newborns undergoing therapeutic hypothermia: a multi-center regional cohort
Source: J Perinatol. 2026 Jul 6;46(9):1801–9. doi: 10.1038/s41372-026-02772-0 (PMC13588749; doi:10.1038/s41372-026-02772-0)
Supplement: Supplementary file 1 [file 41372_2026_2772_MOESM1_ESM.doc]

Neonatal Encephalopathy Score (NES)

|  | Normal  (0 Point each) | Mild/Stage 1  (1 point each) | Moderate/Stage 2  (2 points each) | Severe/Stage 3  (3 points each) |
| --- | --- | --- | --- | --- |
| Spontaneous Activity | 0- Normal |  | 2- Decreased | 3- Absent |
| Level of Consciousness | 0- Normal | 1-Hyperalert/Irritable | 2-Lethargic /Obtunded | 3-Stupor/Coma |
| Muscle Tone | 0- Normal |  | 2- Hypotonic | 3- Flaccid |
| Posture | 0- Normal | 1- Mild Distal Flexion | 2- Strong Distal Flexion | 3- Decerebrate |
| Moro Reflex | 0- Normal | 1- Exaggerated | 2- Weak/Incomplete | 3- Absent |
| Suck Reflex | 0- Normal | 1- Weak | 2- Weak/Incoordinated | 3- Absent |
| Light Reflex | 0- Normal | 1- Dilated | 2- Constricted | 3- Unequal/Fixed dilated |
| Respiration | 0- Normal |  | 2- Periodic Breathing | 3-Apnea |
| Heart Rate | 0- Normal | 1- Tachycardia | 2- Bradycardia | 3- Variable |
| **Total Score** |  |  |  |  |

The NES has a minimum score of 0 and a maximum score of 27. No component is preferentially weighted, and each score in every category is counted.

Brief Neonatal Encephalopathy Score (BNES)

|  | Normal | Mild | Moderate | Severe |
| --- | --- | --- | --- | --- |
| Level of Consciousness  Points | Normal  0 | Hyperalert/Irritable or Sleepy  2 | Lethargic or suspected clinical seizures  3 | Stuporous/Comatose  4 |
| Spontaneous Activity  Points | Normal  0 | Normal  0 | Decreased  2 | Absent  3 |
| Muscle Tone/Posture  Points | Normal  0 | Hypertonic  0 | Hypotonic  2 | Flaccid  3 |
| Moro Reflexes  Points | Normal  0 | Exaggerated  1 | Weak/Incomplete  2 | Absent  3 |
| Respirations  Points | Normal  0 | Normal  0 | Periodic Breathing  2 | Apnea  3 |

The BNES has a minimum score of 0 and an maximum score of 16. It preferentially weights level of consciousness.
